# Supplementary material for: Biochemical Characterization and NMR Study of a PET-Hydrolyzing Cutinase from Fusarium solani pisi
Source: Biochemistry. 2023 Mar 27;62(8):1369–75. doi: 10.1021/acs.biochem.2c00619 (PMC10116592; doi:10.1021/acs.biochem.2c00619)
Supplement: Supplementary file 1 — bi2c00619_si_001.pdf [file bi2c00619_si_001.pdf]

Biochemical characterization and NMR study of a PET-hydrolyzing cutinase from *Fusarium solani pisi*

*Kristina Naasen Hellesnes[1, ‡], Shunmathi Vijayaraj[1, ‡], Peter Fojan[2], Evamaria Petersen[2], Gaston Courtade[1, *]*

[‡] *These authors contributed equally.*

* *Corresponding author: gaston.courtade@ntnu.no*

[1]NOBIPOL, Department of Biotechnology and Food Science, NTNU Norwegian University of Science and Technology, 7491 Trondheim, Norway

[2]Department of Materials and Production, Materials Engineering Group, Aalborg University, 9220 Aalborg Ø, Denmark

SUPPLEMENTARY INFORMATION

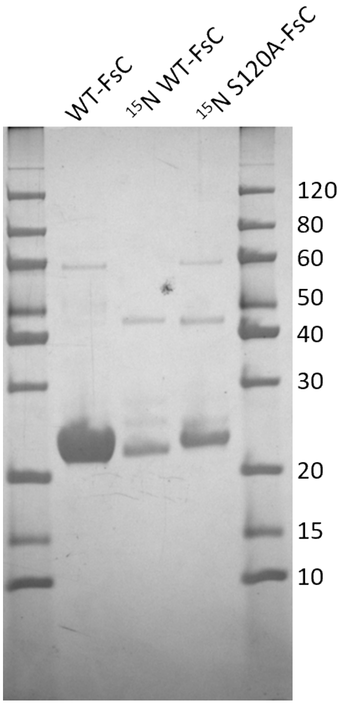

Figure S1. Sodium dodecyl sulphate-polyacrylamide gel electrophoresis (SDS-PAGE). The picture of the stained gel shows the FsC samples used for activity assays (WT-FsC) and NMR titration experiments (15N S120A-FsC), as well as a sample of 15N WT-FsC. The molecular weights (in kDa) of the standards (PAGE-MASTER Protein Standard Plus from GenScript) are indicated for the bands in right-most standard lane.

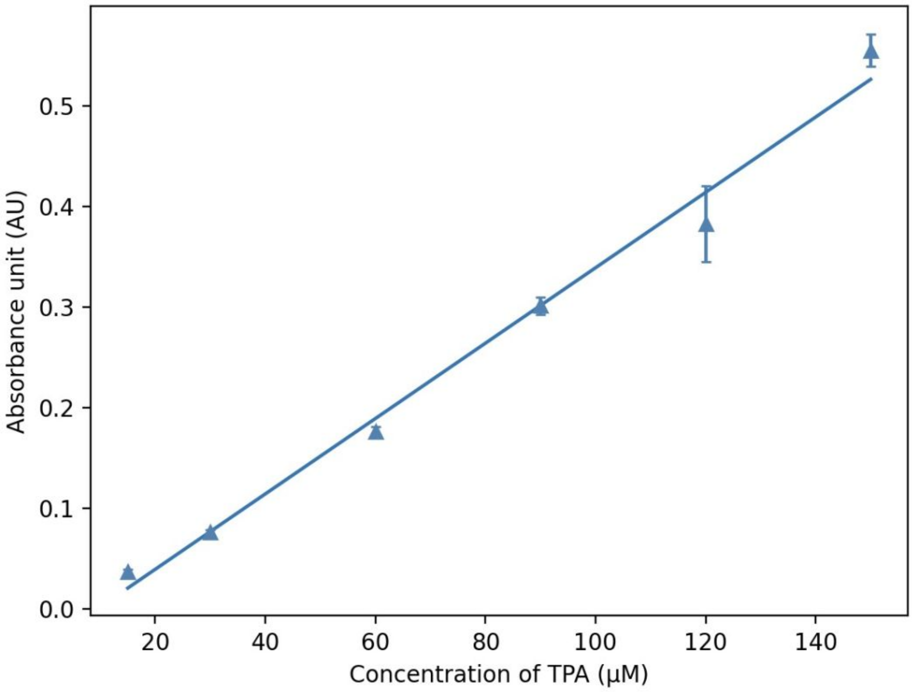

Figure S2. Terephthalic acid (TPA) standard curve. Measured absorbance values at 240 nm as a function of TPA concentration. Linear regression was performed and gave a linear function of A_{240} = 0.0038*[TPA] - 0.0359, with R^2 = 0.99.

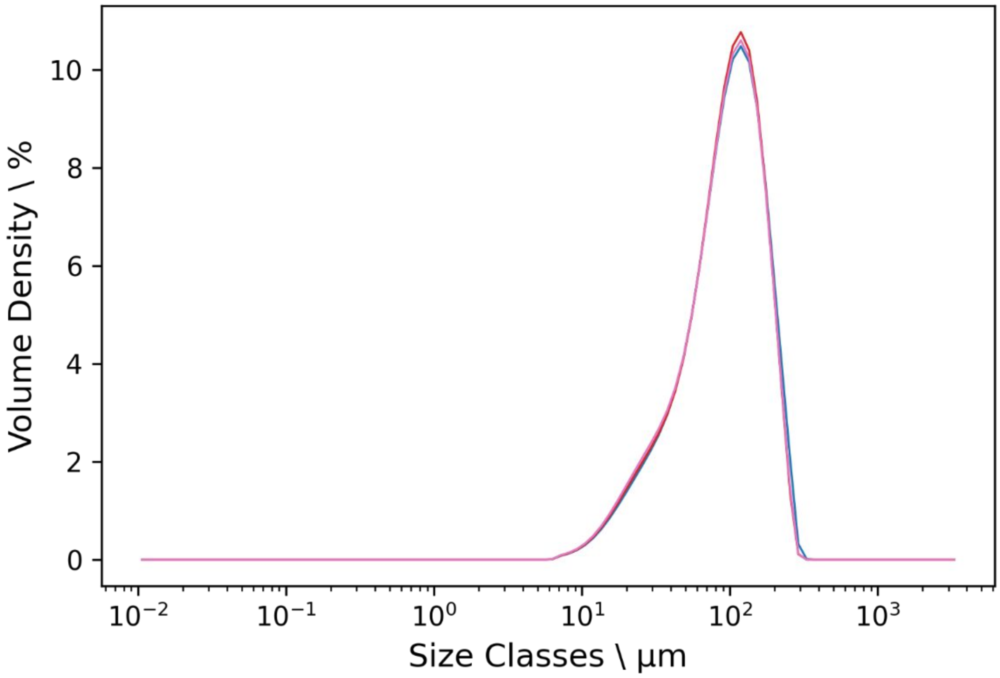

Figure S3. Analysis of particle size distribution of PET powder. The figure shows on overall of five curves describing the distribution of weight classes in a sample of crystalline PET powder (GoodFellow product code ES306031) dispersed in 96% ethanol. The following particle size parameters were determined. Volume-weighted mean diameter, D[4,3] = 103 ± 1 µm; surface area-weighted mean diameter, D[3,2] = 65.3 ± 0.7 µm and specific surface area = 92 ± 1 mm^2 mg^{-1}.

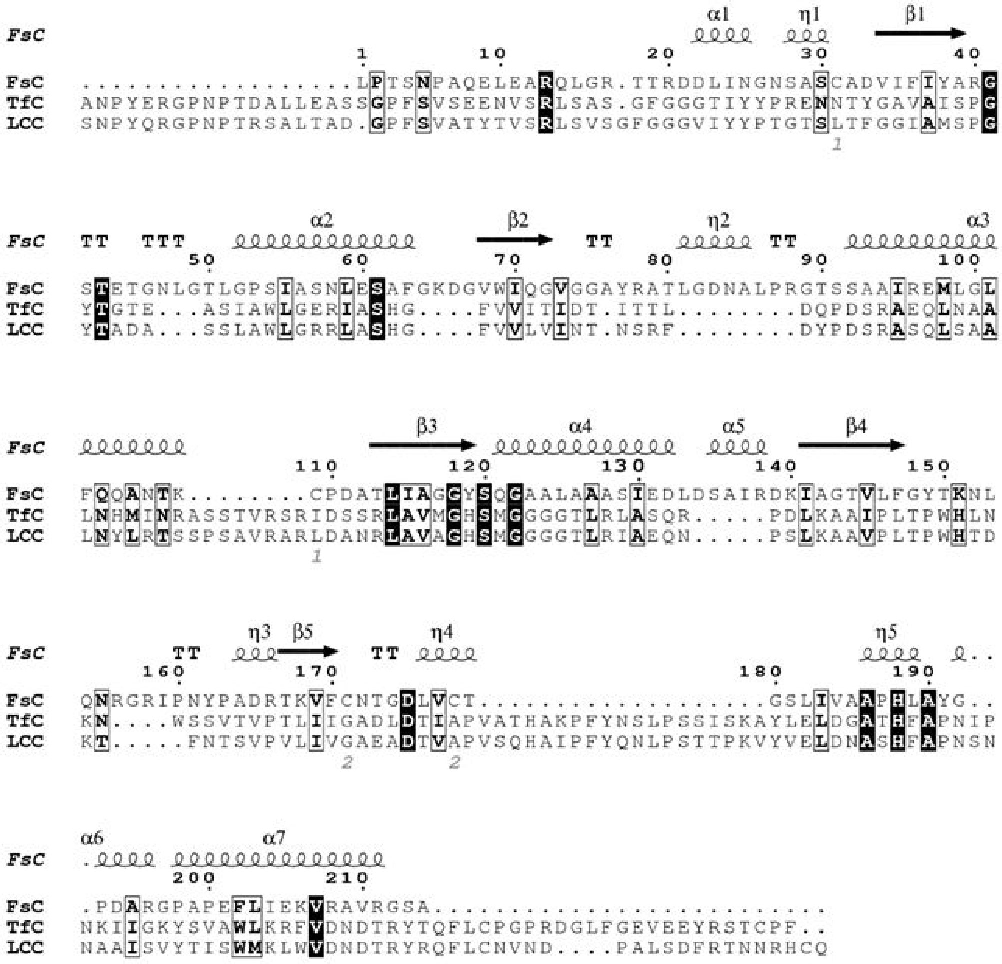

Figure S4. Multiple-sequence alignment (MSA) of three cutinases. The MSA was performed in the Expresso/T-coffee server[1] and the visual representation was made using the ESPript 3.0 server[2]. FsC (*Fusarium solani pisi* cutinase; PDB 1CEX), TfC (*Thermobifida fusca* cutinase; PDB 5ZOA), LCC (leaf-branch compost cutinase; PDB 4EB0). Secondary structure elements from FsC are shown on top (helices with squiggles, β-strands with arrows and turns with TT letters). The bottom numbers (1 and 2) represent the disulphide bonds in FsC. α and η represent alpha- and 3_{10}-helices, respectively. Identical and similar residues are highlighted black and boxed white, respectively.

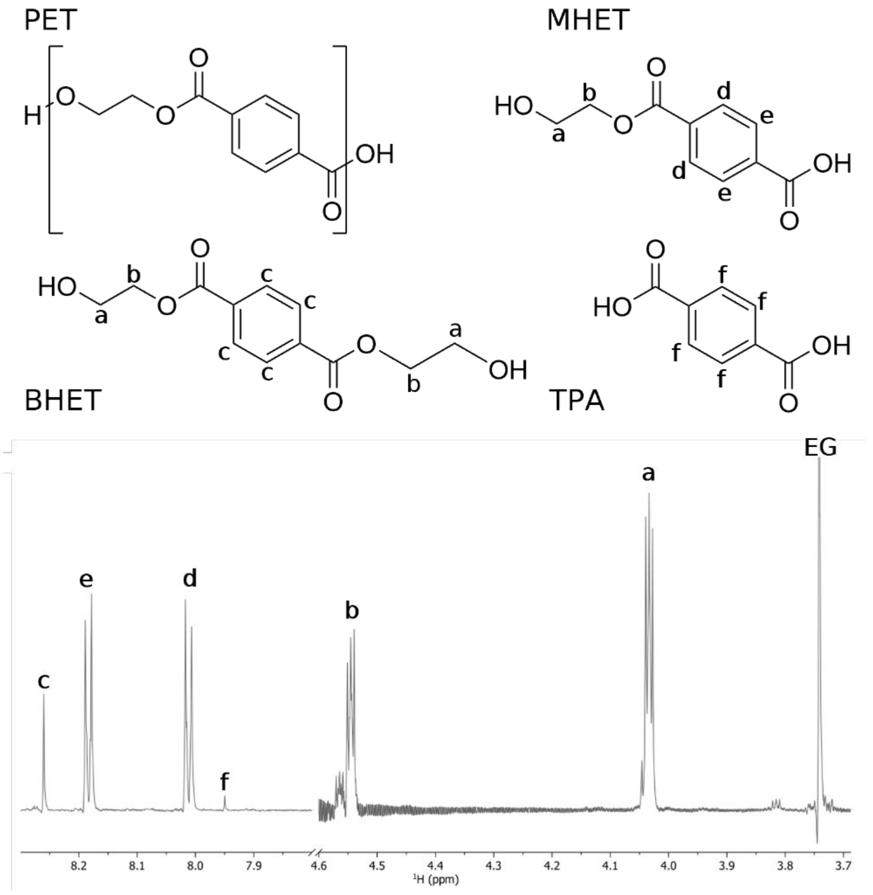

Figure S5. Chemical structures and chemical shifts. The structures of polyethylene terephthalate (PET), bis(2-hydroxyethyl) terephthalate (BHET), mono(2-hydroxyethyl) terephthalic acid (MHET), and terephthalic acid (TPA) are shown. The chemical shifts corresponding to ^{1}H and ^{13}C resonances (in ppm) were assigned at pD 6.5 and 313 K as **a**: 4.02, 59.8; **b**: 4.53, 63.8; **c:** 8.19,129.5; **d:** 8.01, 128.8; **e:** 8.18, 129.5; **f:** 7.94, 129.5. The 1H NMR spectrum under the structures shows an overview of the assigned resonances, as well as the free ethylene glycol (EG) peak at 3.74 ppm.

REFERENCES

(1) Armougom, F., Moretti, S., Poirot, O., Audic, S., Dumas, P., Schaeli, B., Keduas, V., and Notredame, C. (2006) Expresso: automatic incorporation of structural information in multiple sequence alignments using 3D-Coffee. *Nucleic Acids Res. 34*, W604-608.
(2) Robert, X., and Gouet, P. (2014) Deciphering key features in protein structures with the new ENDscript server. *Nucleic Acids Res. 42*, W320–W324.
